# Genetic and epigenetic analyses guided by high resolution whole-genome SNP array reveals a possible role of *CHEK2* in Wilms tumour susceptibility

**DOI:** 10.18632/oncotarget.26123

**Published:** 2018-09-25

**Authors:** Sara Ciceri, Beatrice Gamba, Paola Corbetta, Patrizia Mondini, Monica Terenziani, Serena Catania, Marilina Nantron, Maurizio Bianchi, Paolo D’Angelo, Federica Torri, Fabio Macciardi, Paola Collini, Martina Di Martino, Fraia Melchionda, Andrea Di Cataldo, Filippo Spreafico, Paolo Radice, Daniela Perotti, on behalf of the AIEOP study group

**Affiliations:** ^1^ Molecular Bases of Genetic Risk and Genetic Testing Unit, Department of Research, Fondazione IRCCS Istituto Nazionale Tumori, Milan, Italy; ^2^ Pediatric Oncology Unit, Fondazione IRCCS Istituto Nazionale Tumori, Milan, Italy; ^3^ Department of Hematology and Oncology, Istituto G. Gaslini, Genova, Italy; ^4^ Pediatric Onco-Hematology, Stem Cell Transplantation and Cellular Therapy Division, Regina Margherita Children’s Hospital, Torino, Italy; ^5^ Pediatric Oncology Unit, A.R.N.A.S. Ospedali Civico, Di Cristina e Benfratelli, Palermo, Italy; ^6^ Department of Psychiatry and Human Behavior, School of Medicine, University of California, Irvine, CA, USA; ^7^ Soft Tissue and Bone Pathology, Histopathology, and Pediatric Pathology Unit, Fondazione IRCCS Istituto Nazionale Tumori, Milan, Italy; ^8^ Pediatric Oncology Unit, Pediatric Department, II University, Naples, Italy; ^9^ Pediatric Hematology and Oncology Unit, Bologna University, Bologna, Italy; ^10^ Pediatric Hematology and Oncology Unit, Catania University, Catania, Italy

**Keywords:** Wilms tumor, SNP array, CHEK2

## Abstract

Wilms tumour (WT), the most frequent malignant childhood renal tumour, shows a high degree of genetic and epigenetic heterogeneity. Loss of imprinting on chromosome 11p15 is found in a large fraction of cases and mutations in a few genes, including *WT1*, *CTNNB1*, *WTX*, *TP53* and, more recently, *SIX1, SIX2* and micro RNA processing genes (miRNAPGs), have been observed. However, these alterations are not sufficient to describe the entire spectrum of genetic defects underlying WT development. We inspected data obtained from a previously performed genome-wide single nucleotide polymorphism (SNP) array analysis on 96 WT samples. By selecting focal regions commonly involved in chromosomal anomalies, we identified genes with a possible role in WT development, based on the prior knowledge of their biological relevance, including *MYCN, DIS3L2, MIR562*, *HACE1*, *GLI3*, *CDKN2A* and *CDKN2B*, *PALB2*, and *CHEK2*. The *MYCN* hotspot mutation c.131C>T was detected in seven cases (7.3%). Full sequencing of the remaining genes disclosed 16 rare missense variants and a splicing mutation. Most of these were present at the germline level. Promoter analysis of *HACE1*, *CDKN2A* and *CDKN2B* disclosed partial methylation affecting *HACE1* in a consistent fraction of cases (85%). Interestingly, of the four missense variants identified in *CHEK2*, three were predicted to be deleterious by *in silico* analyses, while an additional variant was observed to alter mRNA splicing, generating a functionally defective protein. Our study adds additional information on putative WT genes, and adds evidences involving *CHEK2* in WT susceptibility.

## INTRODUCTION

Different genetic and epigenetic modifications have been reported in Wilms tumour (WT), a paediatric kidney malignancy [1, 2]. Interestingly, although a loss of imprinting on chromosome 11p15 is found in approximately 70% of cases, each of the genes identified to date appear to be mutated only in a relatively limited subset of cases [3]. *WT1*, the first WT gene to be identified, is involved in no more than 10% of sporadic cases, whereas anomalies affecting the *TP53* gene are restricted to anaplastic WTs. *CTNNB1* mutations, found in approximately 15% of cases, often co-occur with *WT1* mutations, whereas abnormalities of the *WTX* gene on chromosome X were reported to be involved in 7-29% of cases [4, 5]. Even mutations involving the most recently discovered WT genes *SIX1*, *SIX2*, and microRNA processing genes, do not exceed 20% of cases, and often overlap with other genetic aberrations, mainly 11p15 anomalies [6, 7]. Thus, there is a number of sporadic cases in which no mutation in the known WT genes, are found, suggesting that further genes possibly involved in WT etiology, are still to be disclosed.

In the attempt to reduce the number of WT cases with unknown genetic cause, we aimed at using data derived from a high throughput approach, whole-genome SNP array with an average resolution of 8Kb [8, 9], to disclose further genes possibly involved in WT development. In particular, we investigated all detected focal chromosomal anomalies and, after excluding those reported as constitutionally polymorphic (http://projects.tcag.ca/variation/), seven different regions in which genes with a putative role in tumourigenesis and/or kidney organogenesis are mapped, were selected.

## RESULTS

### Selection of chromosome regions and genes

Following the review of the SNP-array data obtained as previously reported [8, 9], chromosome regions and genes involved in focal anomalies were identified. Among these, we selected, through literature data-mining, seven regions harbouring genes with a role in tumourigenesis and/or kidney development (Table 1).

**Table 1:** Selected CN aberrations observed in 96 Wilms tumours (WTs)

Chromosome region	Length of focal anomaly	Focal Anomaly	Number of WTs affected by focal anomaly	Larger anomaly	Number of WTs affected by larger anomaly	Total number of affected WTs (%)	Genes of interest
2p24.3	507kb	CNN^a^ and AI^b^	1 primary	CNN^a^ and AI^b^	5 primary and 1 recurrence	12.5	*MYCN* [8]
		CNG^c^ and AI^b^	4 primary and 1 recurrence				
2q37.1	49kb	HD^e^	2 primary	CNN^a^ and AI^b^	4 primary	7.2	*DIS3L2* and *miR-562* [8]
				CNG^c^ and AI^b^	1 primary		
6q21	1542kb	CNL^d^ and LOH^f^	1 primary	CNG^c^ and AI^b^	1 primary	15.6	*HACE1*
				CNG^c^	1 recurrence		Supplementary Figure 1
				CNL^d^ and LOH^f^	1 primary		
				CNN^a^ and AI^b^	10 primary		
				CNN^a^ and LOH^f^	1 primary		
7p14.1	1325kb	CNL^d^ and LOH^f^	1 primary	CNG^c^ and a AI^b^	1 primary	20.8	*GLI3*
				CNL^d^ and AI^b^	1 primary		Supplementary Figure 2
				CNL^d^ and LOH^f^	8 primary and 1 recurrence		
				CNN^a^ and AI^b^	7 primary and 1 recurrence		
9p21.3	206kb	HD^e^	1 primary	CNL^d^ and AI^b^	1 primary	8.3	*CDKN2A* and *CDKN2B*
				CNN^a^ and AI^b^	4 primary and 1 recurrence		Supplementary Figure 3
				CNN^a^ and LOH^f^	1 primary		
16p12.1	780kb	CNL^d^ and LOH	1 primary	CNL^d^ and LOH^f^	1 primary	6.2	*PALB2*
				CNN^a^ and AI^b^	3 primary		Supplementary Figure 4
				CNN^a^ and LOH^f^	1 recurrence		
22q12.1	994kb	CNN^a^ and AI^b^	1 primary	CNL^d^ and LOH^f^	7 primary and 1 recurrence	13.5	*CHEK2*
				CNN^a^ and LOH^f^	1 primary		Supplementary Figure 5
				CNN^a^ and AI^b^	3 primary		

Abbreviations: ^a^CNN: copy number neutral, ^b^AI: allelic imbalance, ^c^CNG: copy number gain,^d^CNL: copy number loss, ^e^HD: homozygous deletion, ^f^LOH: loss of heterozygosity

Two of these regions 2p24.3, containing the *MYCN* gene, and 2q37, containing the *DIS3L2* and *MIR562* were previously described [8, 9]. On chromosomes 2p24.3 focal overlapping anomalies ranging from 507 to 825 kb were observed in six samples. One primary WT showed allelic imbalance, four primary WTs showed both low level CN (copy number) gain and allelic imbalance. The anomaly observed in one of the latter primaries was detected also in its corresponding recurrent tumour. Further six samples showed larger allelic imbalances on chromosome 2p affecting also the *MYCN* region. In the *DIS3L2* chromosomal region we observed two cases with homozygous deletions on 2q37 occurring within a wider region of loss of heterozygosity (LOH), leading to the complete deletion of the genes *DIS3L2* and *miR-562* [8]. Five other samples showed larger anomalies on chromosome 2q in overlapping regions (allelic imbalance in four cases, CN gain and allelic imbalance in one).

Five additional selected regions were mapped to chromosomes 6q, 7p, 9p, 16p and 22q. In one case, we found focal CN loss and LOH on chromosome 6q21 (chr6:103,951,059-105,493,480), involving only the *HACE1* gene (Supplementary Figure 1). Anomalies spanning *HACE1* were found in additional 14 cases (allelic imbalance and CN gain in one WT, CN gain in one WT, CN loss and LOH in one WT, allelic imbalance in ten WTs and LOH in one WT). In one case, we found focal CN loss and LOH on chromosome 7p14.1 (chr7:41,801,272-43,126,820), involving the *GLI3* gene (Supplementary Figure 2). Other 19 WTs showed larger overlapping chromosomal anomalies on 7p (allelic imbalance and CN gain in one WT, CN loss and allelic imbalance in one WT, CN loss and LOH in nine WTs and allelic imbalance in eight WTs). In one WT sample, we found a homozygous deletion spanning approximately 206kb on chromosome 9p21.3 (chr9:21,806,162-22,012,894), involving the *CDKN2A* and *CDKN2B* genes (Supplementary Figure 3). Additional seven cases showed larger overlapping anomalies on 9p, in particular, CN loss and allelic imbalance in one WT, allelic imbalance in five WTs and LOH in one WT. In one case, we found a focal chromosomal region of CN loss and LOH spanning approximately 780kb on chromosome 16p12.1 (chr16:23,516,852-24,297,082), involving the *PALB2* gene (Supplementary Figure 4). Other five WTs showed bigger anomalies on chromosome 16p affecting the same chromosomal region (CN loss and LOH was detected in one WT, allelic imbalance in three WTs and LOH in one). In one case, we found focal allelic imbalance spanning approximately 994kb on chromosome 22q12.1 (chr22:26,777,048-27,771,791) involving *CHEK2* (Supplementary Figure 5). Other 12 tumours showed larger overlapping anomalies on chromosome 22q (CN loss and LOH were found in eight WTs, LOH in one WT and allelic imbalance in three WTs).

### DNA sequence analysis

The previously reported *MYCN* c.131C>T (p.Pro44Leu) [10] hotspot was found to be mutated in seven out of 96 (ca. 7.3%) WTs.

In the remaining genes (*DIS3L2*, *HACE1*, GLI3, *CDKN2A* and *CDKN2B*, *PALB2* and *CHEK2*), the entire coding region and corresponding intron/exon junctions were sequenced. Excluding common variants, i.e., those reported with a MAF > 0.01 in outbred populations, a total of 25 different alterations were identified, among which 16 missense, eight synonymous and a splice-site variant. Of these, 22 have been already described in genome databases with a MAF < 0.01. Of the three previously unreported variants, two were in *GLI3* and one in *CHEK2* (Table 2).

**Table 2:** Identified variations and *in silico* analyses

Gene	Mutation	Aminoacidic change	het^a^/ hom^b^	Rec^c^	SNP	MAF^d^	*In silico* prediction		
				het^a^/hom^b^			SIFT^e^	Polyphen2^f^	Align-GVGD^g^
*MYCN*	c.131C>T	p.Pro44Leu	7/0		rs1057519919	N.A.^h^	Damaging	Probably Damaging	
*DIS3L2*	c.301G>T	p.Ala101Ser	1/0		rs199857926	1.58e-04	Tolerated	Benign	
*DIS3L2*	c.410A>G	p.Tyr137Cys	1/0		rs201733073	0.001	Tolerated	Benign	
*DIS3L2*	c.1377C>T	p.Ser459=	1/0		rs376722215	3.65e-05			
*DIS3L2*	c.1430T>G	p.Leu477Arg	1/0		rs201719374	6.50e-05	Damaging	Probably Damaging	
*DIS3L2*	c.2424G>A	p.Gln808=	1/0		rs369113667	8.05e-05			
*HACE1*	c.1196A>G	p.Asp399Gly	3/0		rs34365906	0.005	Tolerated	Benign	
*HACE1*	c.1406C>T	p.Pro469Leu	1/0		rs761336527	4.50e-05	Tolerated	Benign	
*GLI3*	c.840C>G	P.Ser280=	1/0		rs77084911	0.002			
*GLI3*	c.1393G>C	p.Gly465Arg	4/1	1/0	rs35488756	0.004	Tolerated	Probably Damaging	
*GLI3*	c.2179G>A	p.Gly727Arg	1/0	1/0	rs121917710	0.005	Damaging	Probably Damaging	
*GLI3*	c.2240C>A	p.Thr747Asn	1/0				Tolerated	Probably Damaging	
*GLI3*	c.2961C>T	p.Tyr987=	1/0		rs528703005	2.80e-05			
*GLI3*	c.4374T>C	p.Gly1458=	1/0						
*GLI3*	c.4554G>C	p.Leu1518=	1/0		rs769537011	4.06e-06			
*CDKN2B*	c.360C>T	p.Ala120=	1/0	1/0	rs62637622	0.001			
*PALB2*	c.768C>T	p.Ser256=	1/0		rs45487491	1.38e-04			
*PALB2*	c.1408A>G	p.Thr470Ala	2/0		rs150636811	1.22e-05	Tolerated	Benign	C0
*PALB2*	c.2794G>A	p.Val932Met	1/0	1/0	rs45624036	0.005	Damaging	Probably Damaging	C0
*PALB2*	c.2816T>G	p.Leu939Trp	1/0		rs45478192	0.001	Damaging	Probably Damaging	C55
*CHEK2*	c.157T>A	p.Ser53Thr	0/1^i^		rs371657037	4.06e-05	Tolerated	Probably Damaging	C55
*CHEK2*	c.410G>A	p.Arg137Gln	2/0		rs368570187	1.79e-04	Tolerated	Benign	C0
*CHEK2*	c.911T>C	p.Met304Thr	1/0		rs587782033	N.A.^**h**^	Tolerated	Probably Damaging	C55
*CHEK2*	c.1095G>T	splicing mut	1/0						
*CHEK2*	c.1312G>T	p.Asp438Tyr	1/0		rs200050883	3.90e-04	Damaging	Probably Damaging	C25

Abbreviations: ^a^het: mutation present in heterozygosity, ^b^hom: mutation present in homozygosity, ^c^Rec: recurrence; ^d^MAF: frequency of the minor allele in gnomAD (ALL); ^e^http://sift.jcvi.org/; ^f^http://genetics.bwh.harvard.edu/pph/; ^g^http://agvgd.hci.utah.edu/agvgd_input.php; ^h^N.A.: not available; ^i^mutation present in hemizygosity.

Four variants [c.1393G>C (p.Gly465Arg) and c.2179G>A (p.Gly727Arg) in *GLI3*, c.360C>T (p.Ala120=) in *CDKN2B* and c.2794G>A (p.Val932Met) in *PALB2*] were identified in tumours that developed recurrence and in all cases were present both in primary and recurrent samples. The c.157T>A (p.Ser53Thr) variant of the *CHEK2* gene was homozygous in the tumour that showed CN neutral LOH in the corresponding chromosomal region.

All variants were found to be present in the constitutional DNA of the patients, with the exception of the c.301G>T (p.Ala101Ser) of *DIS3L2.* The c.1408A>G (p.Thr470Ala) of *PALB2* was detected in two cases, in one of whom at somatic level only.

*In silico* prediction of the consequences of the amino acid changes for the 16 missense mutations are reported in Table 2. Five of them [c.1430T>G (p.Leu477Arg) of *DIS3L2*, c.2179G>A (p.Gly727Arg) of *GLI3*, c.2794G>A (p.Val932Met) and c.2816T>G (p.Leu939Trp) of *PALB2* and c.1312G>T (p.Asp438Tyr) of *CHEK2*] were predicted to be damaging or probably damaging by SIFT and PolyPhen-2. The two latter variants were classified by the Align-GVGD algorithm [11] as scores C55, corresponding to a risk estimate of 0.65 and C25 corresponding to a risk estimate of 0.29, respectively. The *CHEK2* variants c.157T>A (p.Ser53Thr) and c.911T>C (p.Met304Thr) were also scored as C55, but were predicted to be tolerated by SIFT.

**Figure 1:**
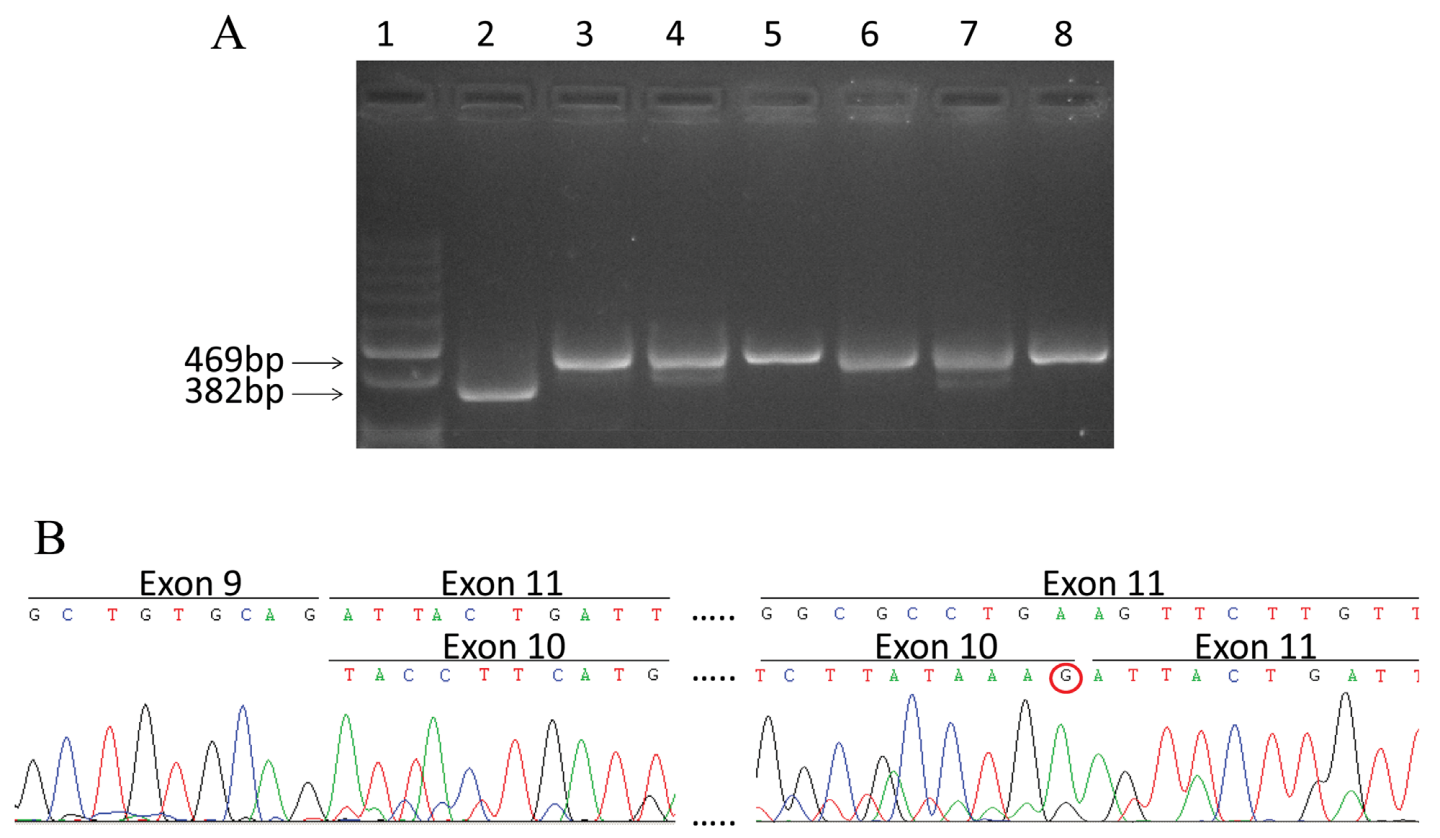
Splicing mutation c.1095G>T in *CHEK2* **(A)** RT-PCR products visualized by agarose gel electrophoresis. Lane 1: Molecular size markers 100bp ladder; lane 2: cDNA from mutated WT; lane 3-8: cDNA from wild type WTs. The size of the full length transcript (469 bp) and Δexon10 transcript (382 bp) are reported. **(B)** cDNA sequence chromatogram of PCR products from the mutated WT, The sequence of Δexon10 and full length transcripts are reported in the upper and lower lane respectively. The absence of the mutated T nucleotide at the last position of exon 10 in the full length transcript (shown by the red circle) indicates that it originates from the wild type allele only.

Among all identified variations, only the novel c.1095G>T variant of *CHEK2* was predicted to affect mRNA splicing by the ALAMUT software. This nucleotide change occurs at the last base of exon 10, and was predicted to cause the loss of the naturally occurring donor splice site. RT-PCR of the region spanning exons 8 to 12 of *CHEK2*, followed by sequence analysis showed the presence, in both the mutant sample and in six control WTs with no *CHEK2* mutations, of the normal (full length; NM_007194) transcript and the naturally occurring mRNA isoform missing exon 10 (Δexon10; NM_145862) (Figure 1). However, in the mutant samples the level of Δexon10 transcript compared to the full-length appeared to be higher than in controls. In addition, the sequencing of the RT-PCR fragment corresponding to the full-length mRNA in the mutated WT revealed the presence of only the transcript form the wild-type allele (Figure 1). These results demonstrate that the *CHEK2* c.1095G>T variant leads to loss of the normal mRNA transcript and the increase of an mRNA isoform carrying the in-frame deletion of exon 10 coding for a protein (p.Tyr337_Lys365del) missing a portion of the protein kinase domain (Supplementary Figure 6).

MSP analysis was performed on all the 96 WTs for the promoter region of *HACE1* and *CDKN2A* and *CDKN2B*. MSP of the CpG-177 island of *HACE1*, previously reported to be frequently methylated in WTs [12, 13], revealed the presence of PCR products from both methylated and unmethylated DNA in 81 tumours (84%), and of the product from unmethylated DNA only in the remaining 14 samples. In eight recurrent tumours, the same methylation patterns detected in the corresponding primary tumours (in seven cases both methylated and unmethylated DNA and in one case unmethylated DNA only) were observed. MSP of the CpG islands in the promoter regions of *CDKN2A* and *CDKN2B* genes revealed the absence of methylation in all samples.

## DISCUSSION

As already detailed, WT is genetically heterogeneous and its pathogenesis is not completely elucidated.

In the present work, we exploited data derived from a whole-genome SNP array analysis to investigate chromosomal anomalies in 96 WTs and disclose genes possibly involved in the development of this malignancy [8].

The rationale of the study was to focus on minimal regions involved in anomalies observed in different tumours, in which to select genes with a putative role in tumourigenesis and/or organogenesis, with relevance to kidney development. Some of the selected genes had been already investigated in WTs. In the following paragraphs, our findings are discussed in detail in the context of available literature data.

Previous studies already observed focal CN gain on 2p24.3 involving *MYCN* in sporadic WTs [8, 14–16]. More recently, *MYCN* CN gains were found in 37/292 (12.7%) WTs, in 26 of which as a focal event [10]. In the present work, focal *MYCN* anomalies were detected in ca. 6% of cases. *MYCN* overexpression has been already reported in WTs, and has been proposed as a possible marker associated with prognosis, as well as with diffuse anaplasia or the presence of blastemal histology after pre-operative chemotherapy [10, 14, 17, 18]. *MYCN* overexpression has been also found in WTs in the absence of chromosomal gain, thus suggesting that other mechanisms (such as hypomethylation at specific regions) may operate to increase the levels of the transcript in a broader range of WTs [10, 19]. A further mechanism causing *MYCN* aberrant activation can be mediated by the hot spot mutation c.131C>T (p.Pro44Leu) initially identified in neuroblastoma as an acquired somatic event with predicted gain of function [20]. This mutation has been recently identified also in a small fraction (ca. 4%) of WTs [7, 10, 21]. In the present series 7% of tumours were found to carry the mutation, further supporting a role of aberrant activation of *MYCN* in WT.

The 2q37 chromosomal region and the *DIS3L2* and *miR-562* genes therein mapped had been already identified as associated with WT development in two congenital syndromic conditions, the 2q37 deletion syndrome and the Perlman syndrome [22, 23]. In fact, three cases with 2q37 deletion syndrome were reported to be affected with WT [reviewed in 22] and, furthermore, *DIS3L2* mutations have been described in Perlman’s syndrome patients [23]. Previous LOH analyses in 226 sporadic WTs identified loss of this region in 4% of cases, in two of which the deletion was homozygous, and within the minimal region commonly lost only the *DIS3L2* and *miR-562* genes were mapped [22]. In the same study, a further 6% of cases showed allelic ratio anomalies in this region, whereas a heterozygous 19-bp deletion of *miR-562* was detected in three out of 176 WTs. However, this deletion is likely to be a neutral variant (rs140596642), with a frequency in non-Finnish Europeans of 1.46%. Finally, no anomalies affecting the sequence of *DIS3L2* were identified in the screening of 96 samples. In a subsequent paper, the sequencing of *DIS3L2* in 40 cases of WTs led to the identification of two missense mutations, whereas MLPA analyses carried out on 20 WTs disclosed the deletion of the entire gene in two cases (in one case the deletion was homozygous) and gene partial deletions, involving only one or few exons, in four cases [23]. In our series, in addition to seven cases with chromosomal abnormalities involving the *DIS3L2* gene previously identified by SNP array analysis, three cases were found to carry *DIS3L2* missense variants, which in two instances were present also at germline level. Whereas the actual impact of such variants on the protein function remains to be assessed, our results are in agreement with previous data indicating a possible involvement of *DIS3L2* in WT development, mainly through deletions.

*HACE1* is a tumour suppressor gene involved in different types of cancers [12]. Its silencing has been shown to be mediated through hypermethylation of a CpG island (CpG-177), located upstream the transcription start site [12, 13]. In WT, genetic anomalies affecting the *HACE1* gene have been previously reported in few cases. In particular, one case displayed a t(6;15)(q21;q21) translocation which led to a lower level of *HACE1* expression [13], whereas another WT showed a 6q21 breakpoint that transected and truncated the *HACE1* gene [24]. Furthermore, two *HACE1* nonsense mutations and six different missense variations have also been reported [7, 24]. Finally, in WTs, previously published data indicated the presence of methylated CpG-177 in ca. 73% of cases and a downregulation of *HACE1* expression, when compared to normal kidney, in 20/26 cases [12]. Among our samples a focal deletion involving solely the *HACE1* gene was observed. In addition, the screening for *HACE1* alterations led to the identification of two germline missense mutations in four samples, indicating a low frequency of gene mutations, in agreement with previous reports, and the investigation of the CpG-177 led to the identification of the presence of methylation in 81/95 (85%) samples, again in agreement with previous data. To the best of our knowledge, this paper describes the first evidence of a WT in which one allele of the *HACE1* gene is deleted, and confirms the proposed role of *HACE1* in WT development, mainly mediated by promoter methylation.

Mutations affecting *GLI3* are responsible of different syndromic conditions, including the Greig cephalopolysyndactyly syndrome (OMIM 175700), the Pallister–Hall syndrome (OMIM 146510) and the Postaxial polydactyly type-A (OMIM 174200), but none of such conditions are associated with WT development. However, a very recent exome sequencing analysis of 58 WT cases has reported nonsense variations of *GLI3* in two WTs [7]. In our samples, *GLI3* sequencing led to the identification of three rare missense variants, also present at germline level, whose impact is unknown, indicating that the role of *GLI3* in WT is still to be established.

Cytogenetic data reported chromosome 9 trisomy in approximately 13% of WTs and LOH studies revealed loss at the *CDKN2A/2B* locus, mapped to 9p21.3 region, in 4/34 (12%) cases [25]. *CDKN2A* mutations have been reported in two children affected with WT in a study assessing the risk of non-melanoma cancers in first-degree relatives of *CDKN2A* mutation carriers [26]. Moreover, a decreased expression of the CDKN2A mRNA isoform encoding the p16^INK4a^ protein was identified in a significant fraction of WTs [27] and correlated with promoter methylation [25, 27–29]. On the contrary, another study reported the absence of methylation of the CDKN2A/p16^INK4a^ promoter in WTs [30]. In our study, mutation screening of *CDKN2A* and the contiguous gene *CDKN2B*, both involved in a homozygous deletion observed in one case, led to the identification of a *CDKN2B* silent variation, already previously reported, and investigation of the promoter regions of *CDKN2A* and *CDKN2B* did not disclose the presence of methylation. While the latter findings argue against a role of *CDKN2A* and *CDKN2B* downregulation in WTs, it must be noted that our study provide the first evidence of a homozygous deletion of *CDKN2A* and *CDKN2B* in WT.

Bi-allelic germline mutations of *PALB2* (also known as *FANCN*) are responsible for the Fanconi anemia (FA) subtype N. Patients affected with this disease show a more severe phenotype compared to other FA subtypes, including growth retardation, variable congenital malformations and predisposition to paediatric malignancies [31]. Among all described carriers of bi-allelic *PALB2/FANCN* mutations who developed cancer in early childhood, four had WT [32, 33]. In addition, a previous study analyzing 47 sporadic WT patients, reported nine germline missense variants in the *PALB2/FANCN* gene, seven of which had been previously reported [31]. Recently, a genome-wide sequencing analysis revealed seven different *PALB2/FANCN* mutations identified in eight of 651 WT patients [34]. In our WT cases, the screening for *PALB2* mutation led to the identification of four previously described rare variants in five patients. Interestingly, two of these variants, [c.2794G>A (p.Val932Met) and c.2816T>G (p.Leu939Trp)] had been previously identified in WT patients [31]. Both variations affect structural residues belonging to a WD 40 domain that binds to the N-terminus of BRCA2 and are predicted to be damaging by both Polyphen2 and SIFT. However, these variations have been also previously described in a familial breast cancer study in which more than 4.000 alleles were screened, and it has been reported to occur with equal frequencies within cases and controls [35]. Therefore, any possible functional role of these variations has yet to be ascertained. Together with previous data, in which *PALB2* anomalies have been rarely identified in WT, with the exception of WTs arising in Fanconi anemia patients, our data suggest that this gene could play a role, if any, in a restricted fraction of sporadic WTs only.

While this study was in progress, germline *CHEK2* mutations in eight of 651 WT patients were reported by Gadd et al. [34]. One mutation (p.Ile157Thr) recurred in four cases and was reported to be likely pathogenic. In the present study, we identified four different *CHEK2* germline missense variants in five patients. Interestingly, three of these variants [c.410G>A (p.Arg137Gln) detected in two cases, c.1312G>T (p.Asp438Tyr) and c.911T>C (p.Met304Thr)] had been previously reported in breast cancer patients [36–38]. In addition, *in vitro* analyses found that the p.Asp438Tyr substitution causes a 70% reduction of kinase activity of the CHEK2 protein [36, 37]. The remaining variant, c.157T>A (p.Ser53Thr) appeared to be reduced to homozygosity in the tumour DNA of the carrier consistent with a two-hit inactivation model of carcinogenesis. Finally, an additional nucleotide change, c.1095G>T, was found to affect RNA splicing. More specifically, this variant induces a relative increase in the expression of a naturally occurring form bearing the deletion of exon 10, and the loss of the full length transcript. It has been reported that the natural isoform of *CHEK2* without exon 10 (Δexon10; NM_145862) loses the kinase activity of the protein [39]. Therefore, our findings suggest a loss of function of the mutant allele.

In summary, this study demonstrates that the use of SNP-array detecting minimal genomic regions commonly involved in anomalies in tumour tissues can be a useful tool to identify genes associated with cancer. In fact, while the clinical significance of the majority of genetic and epigenetic changes we were able to assess remains undetermined, this strategy allowed the identification of loss-of-function mutations in *CHEK2*, a gene previously reported to be involved in susceptibility to cancers of different organs, including breast, colorectum and prostate [40–42]. Therefore, whereas to the best of our knowledge no reports have assessed an increased risk of WT in carriers of germline *CHEK2* mutations, our data, together with those of Gadd and colleagues [34], suggest a putative role of constitutional anomalies of this gene in WT development, which deserves further investigations.

## MATERIALS AND METHODS

### Patients and specimens

The study included material from 96 WTs of 85 patients previously investigated by whole-genome SNP array. In three cases bilateral disease was investigated, and in eight cases both primary and recurrent tumours were studied [8, 9]. The patients represent the first 78 cases prospectively enrolled into the AIEOP WT-2003 protocol for whom tumor samples were available, and seven relapsing cases from the previous AIEOP WT-1992 protocol. A specific informed consent to the use of biological samples for the aim of the study was obtained from the parents or legal guardian of all enrolled patients. The study has been approved by the ethics committee of IRCCS Istituto Nazionale Tumori.

DNA and RNA were purified from frozen surgical specimens from which a histological section was derived and reviewed by the pathologist for the presence of vital tumor tissue.

### Mutation analysis

Sanger sequencing was performed for the *MYCN* hotspot mutation c.131C>T (p.Pro44Leu) [10] and for the entire coding of the *DIS3L2* (Refseq NM_152383), *mir562* (Refseq NR_030288), *CDKN2A* (p14ARF Refseq NM_058195 and p16 Refseq NM_000077), *CDKN2B* (Refseq NM_004936), *HACE1* (Refseq NM_020771), *GLI3* (Refseq NM_000168), *PALB2* (Refseq NM_024675), and *CHEK2* (Refseq NM_007194) genes. Primers and PCR conditions are available upon request.

Variants identified in tumour DNAs were selected if not previously identified or reported with a minor allele frequency (MAF) <0.01 in the gnomAD database linked to ensemble (http://www.ensembl.org/index.html). Selected variants were further investigated in germline DNA. The biological effect of missense mutations was predicted using the Polymorphism Phenotyping v2 (PolyPhen-2; http://genetics.bwh.harvard.edu/pph2/), the SIFT (SIFT; http://sift.jcvi.org/) tools and Align-GVGD (http://agvgd.hci.utah.edu/agvgd_input.php) for the variants identified only in *PALB2* and *CHEK2*. Possible splicing aberrations were analyzed using Alamut (Interactive Biosoftware, Roven, France; http://www.interactive-biosoftware.com/software/alamut/overview).

### Methylation analysis

Bisulfite modification was performed on 300 ng of DNA from WT samples using the EpiTect Bisulfite Kit (QIAGEN, Milan, Italy) according to the manufacturer’s instructions. The methylation status of the promoter of *HACE1*, *CDKN2A* (p14 and p16) and *CDKN2B* (p15) was assessed by methylation-specific PCR (MSP) using primers and conditions already described [12, 27, 43, 44]. Methylated and unmethylated DNAs (QIAGEN, Milan, Italy) were used as positive and negative controls in all MSP reactions.

### Splicing analysis

To determine the effects of c.1095G>T variant on *CHEK2* pre-mRNA processing, total RNA samples were isolated using the RNeasy mini Kit (QIAGEN, Milan, Italy). The cDNAs were prepared using the High Capacity cDNA Archive Kit (Applied Biosystems, Milan, Italy) following the manufacturer’s instructions. The cDNAs were PCR amplified using a forward primer in *CHEK2* exon 8 (5' GATGCAGAAGATTATTATATTGTTTTGG 3') and a reverse primer in *CHEK2* exon 12 (5' GAATGAAGTTGTATTTTCCACTGG 3'). The amplification products were visualized on a 3% agarose gel stained with ethidium bromide and the different PCR fragments characterized by direct sequencing.

## SUPPLEMENTARY MATERIALS FIGURES AND TABLE




