# Supplementary material for: Genetic and epigenetic analyses guided by high resolution whole-genome SNP array reveals a possible role of CHEK2 in Wilms tumour susceptibility
Source: Oncotarget. 2018 Sep 25;9(75):34079–89. doi: 10.18632/oncotarget.26123 (PMC6183341; doi:10.18632/oncotarget.26123)
Supplement: Supplementary file 1 [file oncotarget-09-34079-s001.pdf]

Genetic and epigenetic analyses guided by high resolution whole-genome SNP array reveals a possible role of *CHEK2* in Wilms tumour susceptibility

SUPPLEMENTARY MATERIALS

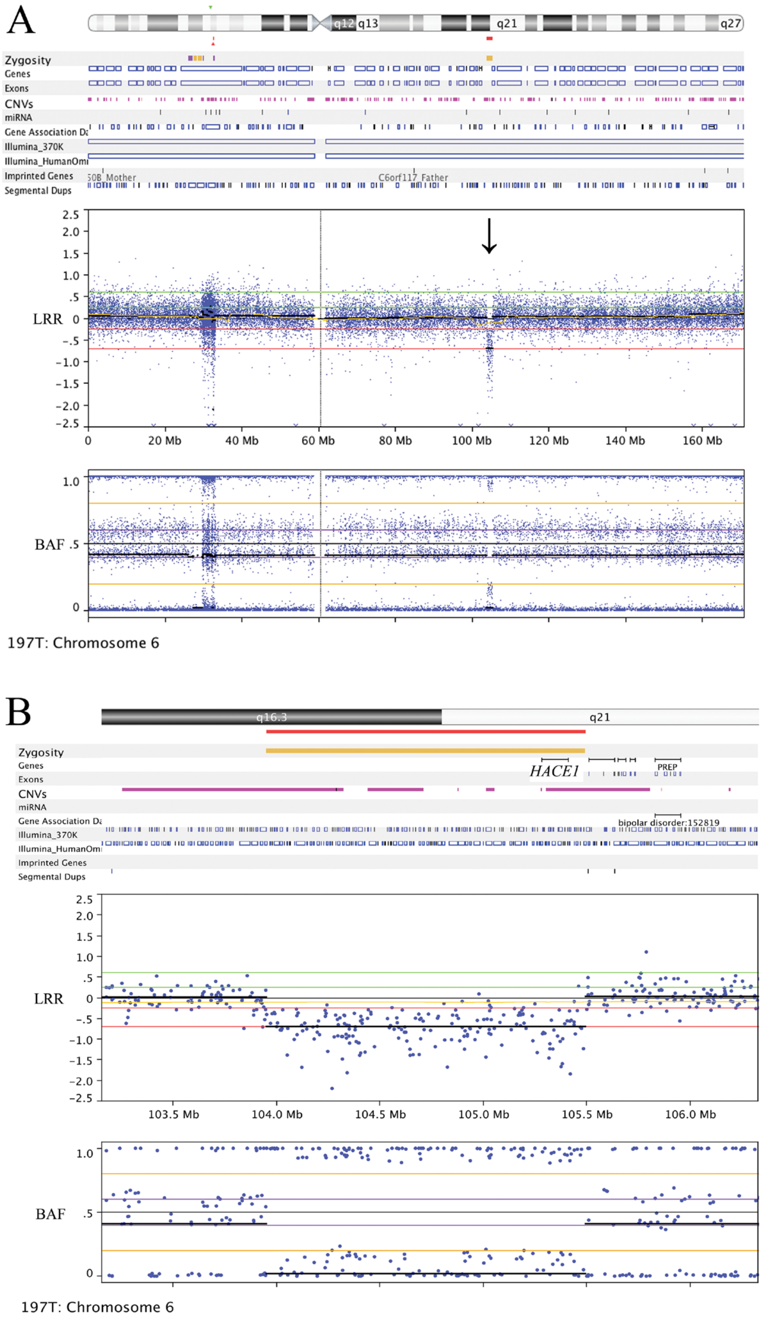

Supplementary Figure 1: Focal 6q21 deletion **(A)** Chromosome 6 ideogram, Log *R* ratio (LRR) and B allele frequencies (BAF) plots of sample WT197. Along the ideogram CN loss is represented by red marks, homozygous copy loss by double red marks, LOH by yellow lines, and allelic imbalance by purple lines. **(B)** Magnification of the 6q21 region, containing the *HACE1* gene, LRR and BAF plots; CN loss and LOH are depicted as detailed in (A).

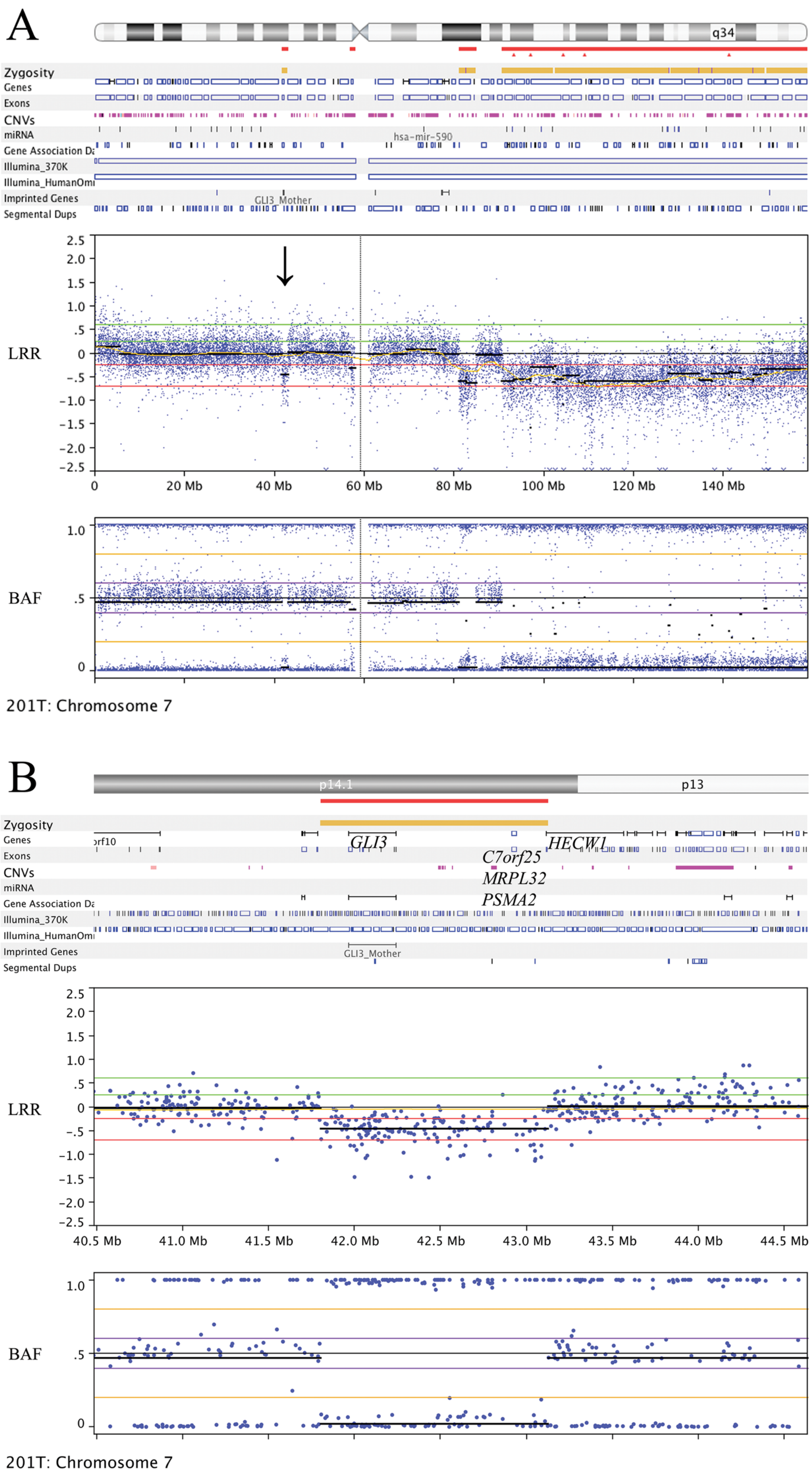

Supplementary Figure 2: Focal 7p14.1 deletion **(A)** Chromosome 7 ideogram, LRR and BAF plots of sample WT201. Along the ideogram CN loss is represented by red marks, homozygous copy loss by double red marks, LOH by yellow lines, and allelic imbalance by purple lines. **(B)** Magnification of the 7p14.1 region, containing the *GLI3* gene, LRR and BAF plots; CN loss and LOH are depicted as detailed in (A).

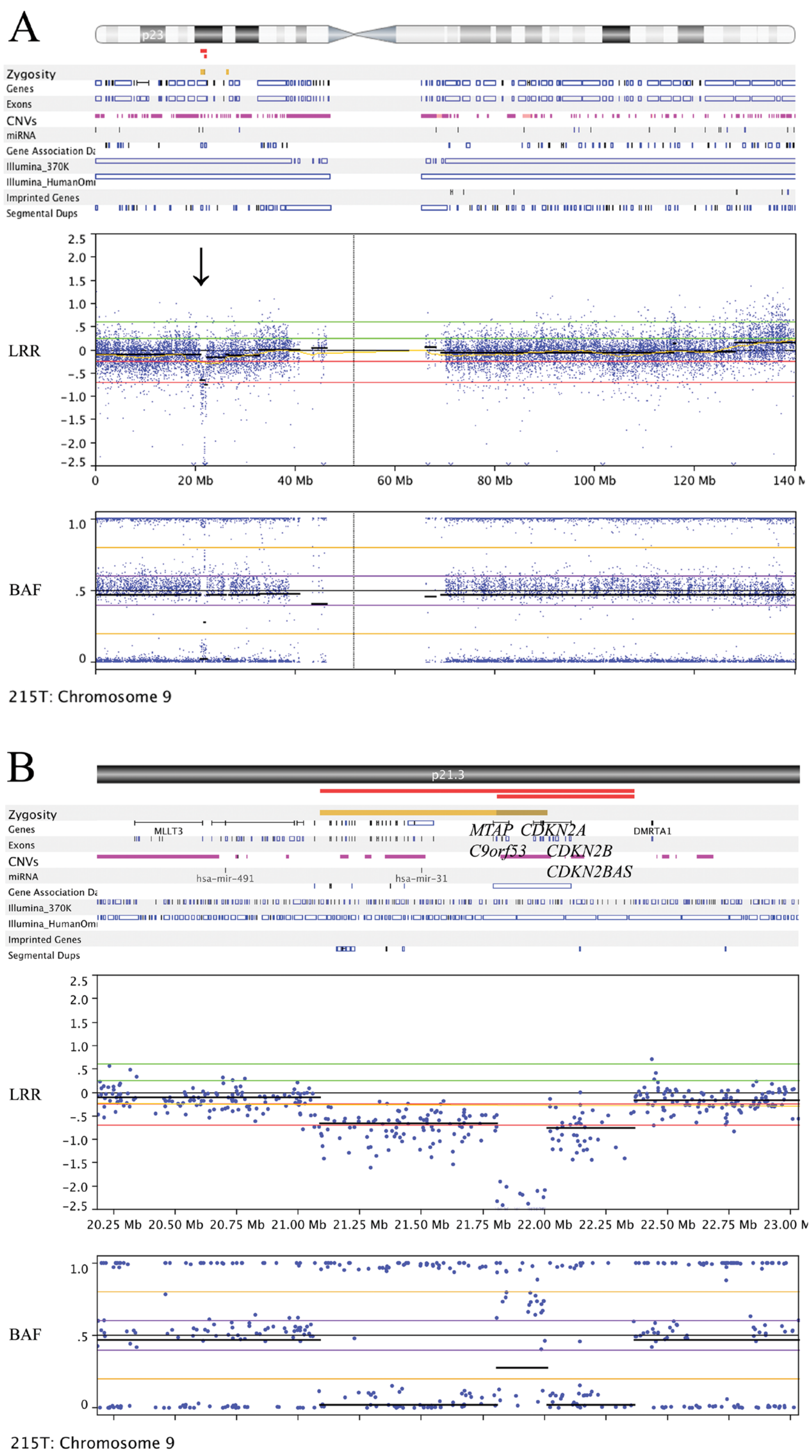

Supplementary Figure 3: Focal 9p21.3 deletion **(A)** Chromosome 9 ideogram, LRR and BAF plots of sample WT215. Along the ideogram CN loss is represented by red marks, homozygous copy loss by double red marks, LOH by yellow lines, and total allelic loss by dark yellow lines. **(B)** Magnification of the 9p21.3 region, containing the *CDKN2A* and *CDKN2B* genes, LRR and BAF plots; CN loss, homozygous copy loss, LOH and total allelic loss are depicted as detailed in (A).

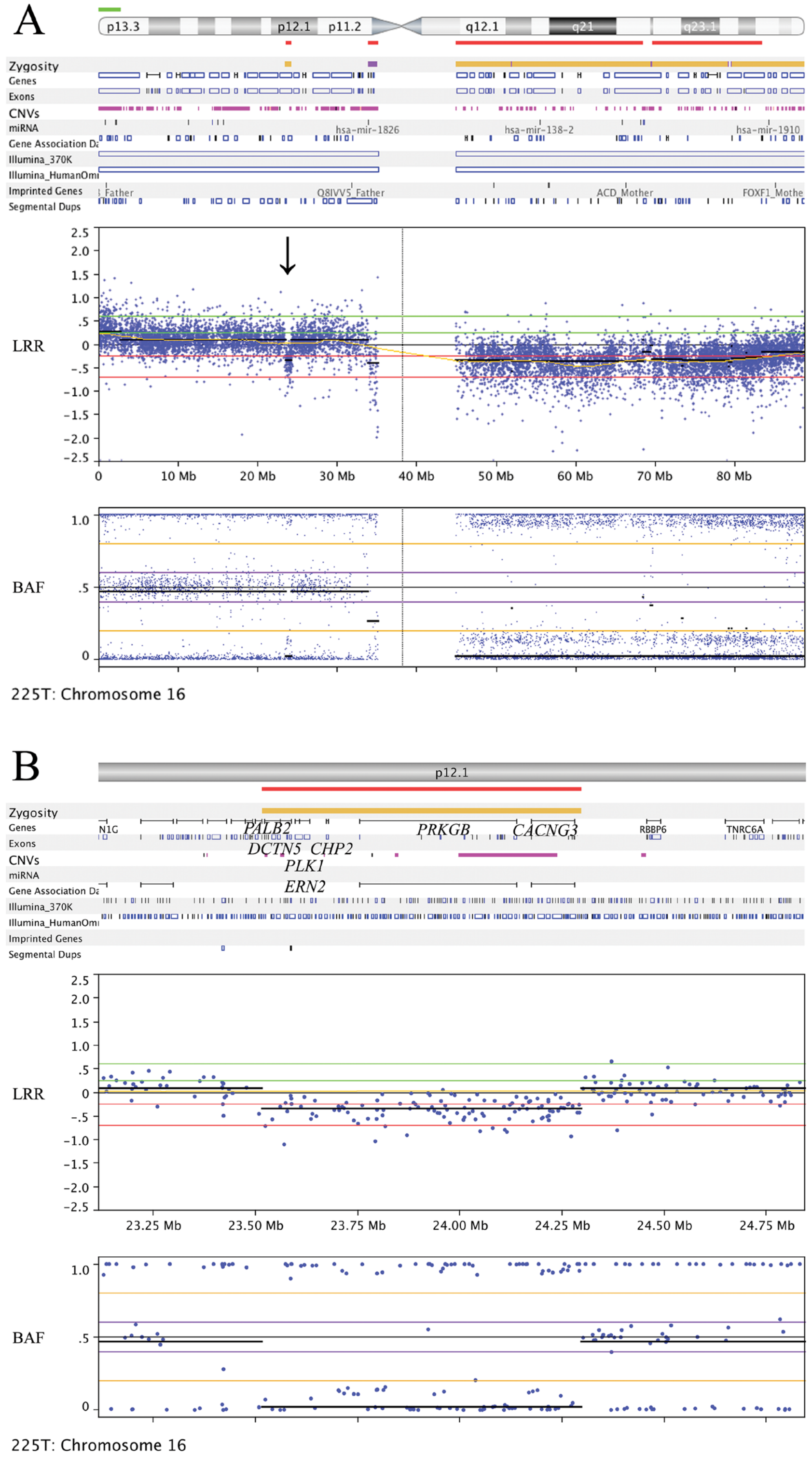

Supplementary Figure 4: Focal 16p12.1 deletion **(A)** Chromosome 12 ideogram, LRR and BAF plots of sample WT225. Along the ideogram CN loss is represented by red marks, CN gain by a green mark, LOH by yellow lines, and allelic imbalance by purple lines. **(B)** Magnification of the 16p12.1 region, containing the *PALB2* gene, LRR and BAF plots; CN loss and LOH are depicted as detailed in (A).

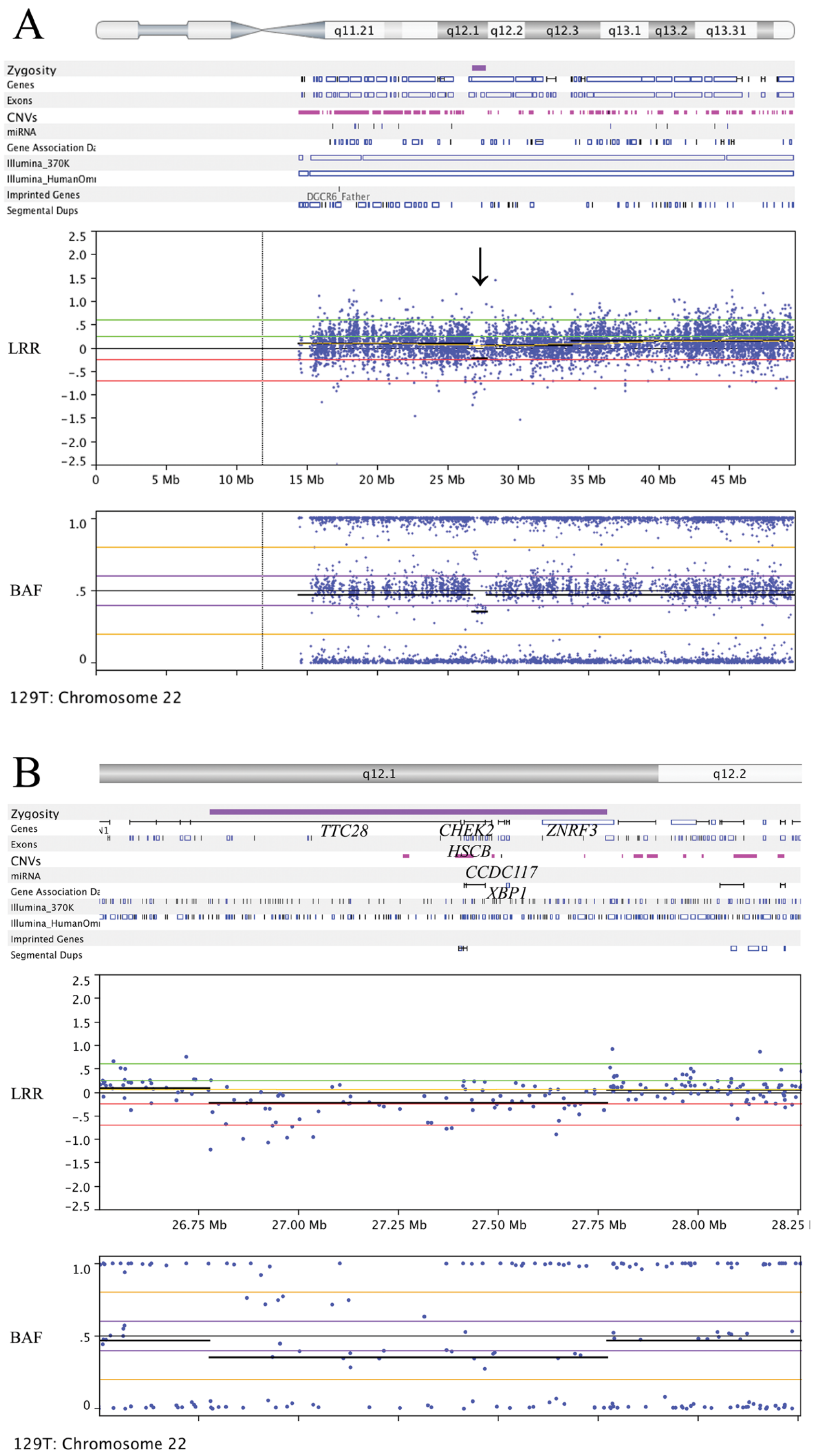

Supplementary Figure 5: Focal 22q12.1 deletion **(A)** Chromosome 22 ideogram, LRR and BAF plots of sample WT129. Along the ideogram allelic imbalance is represented by purple lines. **(B)** Magnification of the 22q12.1 region, containing the *CHEK2* gene, LRR and BAF plots; allelic imbalance is depicted as detailed in (A).

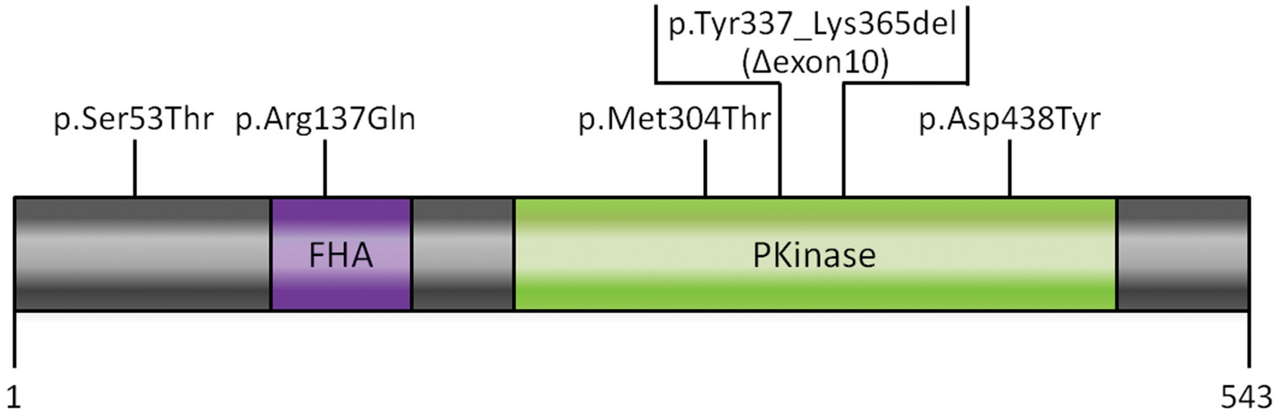

Supplementary Figure 6: Location of *CHEK2* variants within protein domains. The domains present in the protein are indicated by boxes: the FHA Domain (from aminoacid 113 to aminoacid 175, purple box), the Protein kinase Domain (from aminoacid 220 to aminoacid 486, green box).

Supplementary Table 1: Genetic and clinico-pathological data

See Supplementary File 1
